# INFRAVEC: research capacity for the implementation of genetic control of mosquitoes

**DOI:** 10.1179/2047772413Z.000000000174

**Published:** 2013-12

**Authors:** Andrea Crisanti

**Affiliations:** Imperial College, Department of Life Science, London, UK

## Summary

Mosquitoes represent a major and global cause of human suffering due to the diseases they transmit. These include parasitic diseases, i.e. malaria and filariasis, and viral infections such as dengue, encephalitis, and yellow fever. The threat of mosquito-borne diseases is not limited to tropical and subtropical regions of the world. Trade and climate changes have opened new niches to tropical vectors in temperate areas of the world, thus putting previously unaffected regions at risk of disease transmission. The most notable example is the spread of *Aedes* species, particularly the Asian tiger mosquito *Aedes albopictus* to southern Europe (reviewed in Ref. 1). Endogenous cases of vector-borne diseases including West Nile fever, chikungunya, and dengue are frequently being reported, highlighting the increased risk of tropical diseases for the European population.^2^–^5^

Typically, vector control measures targetting mosquitoes are in most cases carried with the use of insecticides. This approach has a number of limitations that constrain their effectiveness. Lack of resources, inadequate logistics, and the insurgence of insecticide resistance are some of the problems encountered in disease-endemic countries (DECs). More recently in Africa, the widespread use of insecticide-treated bed nets has caused a dramatic reduction in malaria mortality and morbidity.^6^ Bed nets however are a temporary solution, a testimony of the failure to implement area-wide control measures aimed at eradicating malaria. US and Europe, with well-developed economies, have also failed to control the spread of mosquito vectors, particularly *Aedes* species.^1^ This alarming situation clearly speaks for the need to expand the knowledge on mosquito vectors and for the urgency of developing and validating novel biological and genetic control measures that overcome the limitations of current insecticide-based approaches. During the last 10 years, significant advances have been made in understanding the biology, the genetics, and the ecology of *Anopheles* and *Aedes* mosquitoes paralleled by the development of new molecular tools for investigating gene function and mosquito ability to transmit parasite and viral diseases. They offer a compelling opportunity to design and validate new genetic vector control measures. The size and the complexity of this undertaking require a high level of capacity, effort, and technological platforms. No laboratory – or even institution – has the resources, the infrastructure capacity, and the expertise to accomplish this task alone.

INFRAVEC addresses the need of the scientific community to share facilities and integrate cutting-edge knowledge and technologies that are not readily accessible but nevertheless critical to exploit genetic and genomic information in the effort to control mosquito-borne diseases. Its objective is to provide laboratories that currently operate individually with limited coordination and little sharing of technologies, with the collective research capacity of the laboratories forming the core project infrastructure. INFRAVEC has provided resources to 31 institutions from European and African countries to enhance collaborative links, to execute joint research activity, and most importantly to enable individual researchers (from PhD students to established academics) to carry complex experimental activities by assigning research packages or ‘infrastructure access’ to be executed in the laboratory facilities and infrastructures of INFRAVEC. I report here on the overall activities of INFRAVEC and its impact on the scientific community with the purpose to initiate a dialogue with all stakeholders on its future evolution.

## Project Structure

European laboratories have made crucial contributions in the field of mosquito molecular biology, genetics, biology, and epidemiology and have established a number of valuable facilities to perform insect mass rearing, mosquito genetic manipulation, single nucleotide polymorphism (SNP) population typing, and confined release experiments. These laboratories have so far mainly operated individually with limited coordination and little sharing of infrastructure and technologies. INFRAVEC has the objective to integrate these facilities into a coordinated infrastructure to promote scientific exchanges, facilitate the sharing of technological platforms, and enhance the research capacity of the scientific community. Its activities are articulated in networking, joint research projects, and promoting and supporting the use of the facility infrastructure by individual researchers. Networking activities have been aimed at improving and utilizing the facilities of the infrastructure as well as at strengthening and facilitating collaborative projects between institutions to improve, assess, and validate genetically manipulated mosquitoes for vector control. They include the development and maintenance of common facilities, such as the formation of a mosquito line repository facility by coordinating different laboratories to stock naturally occurring and genetically modified (GM) mosquito lines together with the formation of a database containing genetic and biological information of each line. 

INFRAVEC has supported four joint research and development activities aimed at (i) improving mosquito gene manipulation technologies, (ii) targetting the mosquito vectorial capacity, (iii) analyzing population structure and gene flow in mosquito species, and (iv) validating GM mosquitoes for vector control under confined field conditions. These projects were leveraged by access to the infrastructure with a range of complementary facilities to provide the research projects with a formidable capability to undertake complex experimental activities. The infrastructure of INFRAVEC consists of four laboratory facilities based in different European countries including (i) the insect mass rearing facilities at the Centro Agricoltura Ambiente in Bologna (Italy), (ii) the mosquito genetic manipulation laboratory at Imperial College London (UK), (iii) the mosquito confined release facility at the Biotechnology Centre of Terni – University of Perugia (Italy), and (iv) the bioinformatics suite for data collection software development and information sharing at EMBL-EBI Cambridge (UK). Some of these facilities such as the bioinformatics suite at EMBL-EBI are widely utilized by the scientific community, whereas others offer their services sporadically and without coordination. INFRAVEC aims at integrating this infrastructure with networking activities and joint research projects to serve the mission of overcoming the existing capacity roadblock on the way to assess ethical, safety, efficacy, and feasibility issues of genetically manipulated mosquitoes for vector control.

INFRAVEC supports individual researchers to carry out research packages in the infrastructure facilities operated by the participants. The infrastructures are made available to external researchers or teams via a grant responsive mode. INFRAVEC publicizes the services offered by the infrastructure to external users and implements a peer review selection process to identify the projects that will be given support to utilize the infrastructure and contribute to the further development of the joint research activities.

## Impact on the Scientific Community

INFRAVEC has been crucial for consolidating European leadership in the field of vector biology, for progressing towards the development of novel genetic control measures and for assisting African laboratories in their effort to develop independent research profiles. This has been achieved by providing resources for implementing research projects, for establishing collaborative links, and for supporting individual researchers to carry out projects in the laboratories forming the project infrastructure facilities. A number of important scientific advances have been achieved and published on more than 30 articles featuring on high impact scientific journals. The following is a non-exhaustive list of publications ordered by journal impact factor.

**Table:** 

Journal	Publication title
PLoS Biology	The interaction between a sexually transferred steroid hormone and a female protein regulates oogenesis in the malaria mosquito *Anopheles gambiae*
Proceedings of the National Academy of Sciences, USA	Wolbachia strain wMel induces cytoplasmic incompatibility and blocks dengue transmission in *Aedes albopictus*
Blood	A switch in infected erythrocyte deformability at the maturation and blood circulation of *Plasmodium falciparum* transmission stages
Current Opinion in Microbiology	*An. gambiae* pathogen susceptibility: the intersection of genetics, immunity, and ecology
	Mosquito/microbiota interactions
Environmental Microbiology	The yeast *Wickerhamomyces anomalus* (*Pichia anomala*) inhabits the midgut and reproductive system of the Asian malaria vector *Anopheles stephensi*
Trends in Parasitology	The invasive mosquito species *Ae. albopictus*: current knowledge and future perspectives
PLoS Neglected Tropical Diseases	Female-specific flightless (fsRIDL) phenotype for control of *Ae. albopictus*
PLoS ONE	*Culex pipiens*, an experimental efficient vector of West Nile and Rift Valley fever viruses in the Maghreb region
	Identification of the midgut microbiota of *An. stephensi* and *Anopheles maculipennis* for their application as a paratransgenic tool against malaria
	High efficiency of temperate *Ae. albopictus* to transmit chikungunya and dengue viruses in the southeast of France
	Dissemination and transmission of the E1-226V variant of chikungunya virus in *Ae. albopictus* are controlled at the midgut barrier level
	Immunogenic and antioxidant effects of a pathogen-associated prenyl pyrophosphate in *An. gambiae*
FEMS Microbiology Ecology	Midgut bacterial dynamics in *Aedes aegypti*
Journal of Evolutionary Biology	The impact of uniform and mixed species blood meals on the fitness of the mosquito vector *An. gambiae* s.s.: does a specialist pay for diversifying its host species diet?
Malaria Journal	Comparative analyses reveal discrepancies among results of commonly used methods for *An. gambiae* molecular form identification
	Wide cross-reactivity between *An. gambiae* and *Anopheles funestus* SG6 salivary proteins supports exploitation of gSG6 as a marker of human exposure to major malaria vectors in tropical Africa
	The impact of low erythrocyte density in human blood on the fitness and energetic reserves of the African malaria vector *An. gambiae* s.s.
	High-throughput sorting of mosquito larvae for laboratory studies and future vector control interventions
Parasites & Vectors	Molecular evidence of *C. pipiens* form molestus and hybrids pipiens/molestus in Morocco, North Africa
	Asaia–anopheles immune system and asaia–plasmodium interactions: perspectives in the control of malaria infection
Insect Biochemistry and Molecular Biology	The *An. gambiae* cE5, a tight- and fast-binding thrombin inhibitor with post-transcriptionally regulated salivary-restricted expression
Microbial Biotechnology	Microbial symbionts: a resource for the management of insect-related problems
BMC Microbiology	Horizontal transmission of the symbiotic bacterium *Asaia* sp. in the leafhopper *Scaphoideus titanus* Ball (Hemiptera: Cicadellidae)
	Delayed larval development in Anopheles mosquitoes deprived of *Asaia* bacterial symbionts
European Journal of Clinical Microbiology and Infectious Diseases	Do mosquito-associated bacteria of the genus *Asaia* circulate in humans?
Pesticide Biochemistry & Physiology	Insecticide resistance in the major dengue vectors *Ae. albopictus* and *Ae. aegypti*
Antonie Van Leeuwenhoek	Different mosquito species host *W. anomalus* (*P. anomala*): perspectives on vector-borne diseases symbiotic control
Journal of Medical Entomology	Mating competitiveness of *Ae. albopictus* radio-sterilized males in large enclosures exposed to natural conditions
	Efficiency of three diets for larval development in mass rearing *Ae. albopictus* (Diptera: Culicidae)
Journal of Applied Entomology	Mosquito symbioses: from basic research to the paratransgenic control of mosquito-borne diseases
Journal of Entomological and Acarological Research	Molecular typing of bacteria of the genus *Asaia* in malaria vector *Anopheles arabiensis* Patton,1905
Pathogens and Global Health	Symbiotic control of mosquito-borne disease

Crucial progresses have been made towards the objective of understanding at the genetic level the population structure of *Anopheles gambiae* mosquitoes culminating with the sequencing of and M and S genetic forms and the identification of previously unrecognized species. New sophisticated bioinformatics approaches have been developed to keep pace with unprecedented developments in sequencing technologies. New gene manipulation technologies have been developed that allow the selective targetting of mosquito genome sequences and the engineering of large mosquito populations. A confined release facility that can closely reproduce field environmental conditions in terms of light exposure, humidity, and temperature – including stochastic climate variability – has been utilized to assess the ecology of both field and genetically manipulated mosquitoes. A total of 47 research projects have been granted access involving analysis of mosquito genetics, genome sequencing, bioinformatics, and gene manipulation to facilitate the sharing of technological platforms and enhance the research capacity of individual laboratory research. Access, measured in ‘units’ or in ‘activity packages’ (a collection of units), includes logistical, technological, and scientific support in the form of either service or specific training that is normally provided to external researchers using the given infrastructure. The table below details the units awarded and the facility offered to them.

**Table:** 

Unit of access available	INFRAVEC facility	Units awarded
Genetic screening	The Malaria Centre (Imperial College London)	6
Embryo microinjection		4
Genetically manipulated mosquitoes		4
RNA extraction		7
Microarray hybridisation experiments		6
High-throughput sequencing		18
Bioinformatic analysis of transcriptomes	The Malaria Centre – SNP Suite (Imperial College London)	8
SNPs analysis		2
Mid- and high-level production of *Aedes albopictus*	Mass Rearing Laboratory (Centro Agricoltura e Ambiente G. Nicoli)	5
Bioinformatic access	European Bioinformatics Institute (EMBL)	6
Database analysis		1
Sorting of mosquitoes	Confined Release Laboratory (University of Perugia)	1
Confined medium- and large-scale release		1

INFRAVEC has also promoted the formation of a mosquito line repository facility by coordinating different laboratories to stock, in their insectaries, naturally occurring and genetically modified mosquito lines together with the formation of a database containing genetic and biological information of each line. Coordination ensures that agreed standards have been implemented across different laboratories and that external users may have access to a unique collection of mosquito lines upon request through the INFRAVEC website. INFRAVEC has also supported the development of a high-throughput technology for the automated sorting of large numbers of mosquitoes. A sorting instrument has been developed in collaboration with Union Biometrica Inc. to separate hundreds of thousands of mosquito larvae daily on the basis of the expression of fluorescent markers in tissues and organs. This technology has proven extremely useful to dramatically increase the yield of mass rearing and the throughput of genetic screens.

## Future Development

In response to a call for letters of intent published last year by the EU infrastructure programme, the principal investigators of the INFRAVEC core facilities have presented a proposal for renewal. The new proposal, while maintaining the overall INFRAVEC architecture combining infrastructure facilities, for transnational access and services, with networking and research activities, for integrating the efforts of the research community, brings important novelties that take into account the development of new technologies, the scientific progress, and the enhanced research capacity of the scientific community. Additional facilities have been included that provide an enhanced and unique research capacity for (1) investigating the ability of mosquito species to transmit a variety of viral and parasitic diseases, (2) performing new cost-effective mosquito genotyping analysis and phenotypic associations, (3) accessing next-generation sequencing technologies, and (4) carrying out release studies under confined conditions. Such enhanced infrastructure capacity will be mobilized towards the development of networking and research activities that have the objective to (1) generate a European risk map for viruses potentially transmitted by *Aedes* mosquitoes, (2) validate the cost effectiveness and the safety of GM mosquitoes for vector control, and (3) investigate how the interaction of mosquitoes with environment microbiota shapes their vectorial capacity.

Networking activities will involve the strengthening of the existing multicentre mosquito repository to include the phenotypic and genotypic characterization of more than 100 mosquito lines of different genetic backgrounds as well as their genome sequencing. A database will also be created to facilitate the association of genotypic variant with biological relevant phenotypes. The project's main mission will again focus on providing resources to researchers at all levels of their career in a bottom-up response mode to carry out experimental activities in the core facility laboratories in the form of self-contained research packages or ‘access units’. Access units or research packages will focus on two mosquito species of great medical and environmental relevance: the human malaria vector *An. gambiae*, and the world’s most invasive species, *Ae. albopictus*, which can function as a vector for a variety of viral diseases, including dengue fever and chikungunya.

The wider scientific community will access the following facilities: (1) the high security P3 insectary of Institut Pasteur for investigating the ability of mosquito species and strains from different genetic backgrounds and/or experimentally manipulated to transmit a number of parasitic and viral diseases; (2) the next-generation sequencing facility of the University of Perugia for analyzing the transcription repertoire of mosquito tissues and organs, and sequencing laboratory and field mosquito genomes; (3) the bioinformatic suite of EMBL-EBI for acquiring expertise in software development and data analysis, and extracting functional information from sequence data. This will also involve a helpdesk to follow the users in their bioinformatic learning curve, including on-site visits and consultations; (4) the genetic laboratory at Imperial College to genetically engineer mosquitoes for assessing gene function, and develop mosquito lines suitable for genetic vector control; (5) the confined release facility at the University of Perugia to assess and validate, under conditions that reproduce the field environment, the safety and effectiveness of genetically manipulated mosquitoes for vector control; and (6) the field facilities of International Centre of Insect Physiology and Ecology to study the ecology and the behaviour of mosquito strains in natural and controlled environments. This combination has no equal in the world scientific landscape. It will offer the research community an unprecedented research power well beyond individual capacities, and will place Europe in the forefront of global research and translation on vector-borne human disease. In theory, a single project may include complex experimental workflow involving genetic transformation of mosquito species, their phenotypic analysis in semi-field conditions, and functional transcription analysis. The overall objective is to progressively transform research activities in commodities and change the way research is carried out from time consuming and laboratory intensive to mainly conceptual methods.
